# The mechanism and therapy of aortic aneurysms

**DOI:** 10.1038/s41392-023-01325-7

**Published:** 2023-02-03

**Authors:** Jianing Gao, Huanhuan Cao, Gaofei Hu, Yufei Wu, Yangkai Xu, Hongtu Cui, Hong S. Lu, Lemin Zheng

**Affiliations:** 1 The Institute of Cardiovascular Sciences and Institute of Systems Biomedicine, School of Basic Medical Sciences, Key Laboratory of Molecular Cardiovascular Science of Ministry of Education, NHC Key Laboratory of Cardiovascular Molecular Biology and Regulatory Peptides, Beijing Key Laboratory of Cardiovascular Receptors Research, Health Science Center, Peking University, 100191 Beijing, China; 2 Department of Cardiology and Institute of Vascular Medicine, Peking University Third Hospital, NHC Key Laboratory of Cardiovascular Molecular Biology and Regulatory Peptides, Key Laboratory of Molecular Cardiovascular Science, Ministry of Education, Beijing Key Laboratory of Cardiovascular Receptors Research, Research Unit of Medical Science Research Management/Basic and Clinical Research of Metabolic Cardiovascular Diseases, Chinese Academy of Medical Sciences, 100191 Beijing, China; 3 Department of Physiology, Saha Cardiovascular Research Center, University of Kentucky, South Limestone, Lexington, KY 40536-0298 USA

**Keywords:** Cardiovascular diseases, Cardiology

## Abstract

Aortic aneurysm is a chronic aortic disease affected by many factors. Although it is generally asymptomatic, it poses a significant threat to human life due to a high risk of rupture. Because of its strong concealment, it is difficult to diagnose the disease in the early stage. At present, there are no effective drugs for the treatment of aneurysms. Surgical intervention and endovascular treatment are the only therapies. Although current studies have discovered that inflammatory responses as well as the production and activation of various proteases promote aortic aneurysm, the specific mechanisms remain unclear. Researchers are further exploring the pathogenesis of aneurysms to find new targets for diagnosis and treatment. To better understand aortic aneurysm, this review elaborates on the discovery history of aortic aneurysm, main classification and clinical manifestations, related molecular mechanisms, clinical cohort studies and animal models, with the ultimate goal of providing insights into the treatment of this devastating disease. The underlying problem with aneurysm disease is weakening of the aortic wall, leading to progressive dilation. If not treated in time, the aortic aneurysm eventually ruptures. An aortic aneurysm is a local enlargement of an artery caused by a weakening of the aortic wall. The disease is usually asymptomatic but leads to high mortality due to the risk of artery rupture.

## Discovery of aneurysms

Galen is credited with being the first to define and describe aneurysms, mainly those that are visible and palpable on the surface of the body, while deep-seated aneurysms are the most common. In the 2nd century, Antyllos was a pioneer in the field of aneurysm surgery and discovered the differences between false and true aneurysms. Aetius, in the 6th century, described the clinical symptoms of aneurysms and revealed that they could occur anywhere in the body, even the head. Before the 16th century, aneurysm research seems to have stalled. Aortic aneurysms (AA) were first described by Saporta in 1554 and first diagnosed by Vesalius the following year. Subsequently, from the 17th century to the 18th century, many scholars explored the pathological features and etiology of aneurysm formation, mainly including the following: (1) Sennertus in 1628 believed that it was the rupture of the inner coat that led to the expansion of the external coat; (2) Iseman in 1676 dismissed this idea, arguing that aneurysms were formed mainly because of a rupture in the aortic wall that allows blood to diffuse to extravasation tissues; and (3) Bourdelot discovered nontraumatic aneurysms in 1681, but aneurysms formed by external trauma remained the most common and familiar. Lancisi, in 1728, was the first to mention a congenital vascular defect as a possible cause of vessel dilation under pressure. Alexander Monro in 1733 described the difference between true and false aneurysms in forming vascular wall damage but mistakenly believed that true aneurysm was rare and did not recognize the phenomenon of elastic tissue being replaced by fibrous tissue in the early stages of an aneurysm. Home and John Hunter believed that preexisting arterial disease was the cause of aneurysm. Donald Monro, reflecting on a macroscopic dissection in 1760, said that aneurysms were not caused by the rupture of the inner layer and the expansion of the outer layer but by the expansion of all layers. In the 19th century, due to the advent of the microtome and microscope as well as the pathologist, the world had a deeper understanding of the pathological mechanism of aneurysm. Welch isolated syphilis from aneurysmal tissue in 1875, thinking that syphilis was an additional factor in the etiology of aneurysms. Coats and Auld in 1893 discovered early aneurysms developed by the blood passing through atheromatous ulcers, and elastic fibers were found to break suddenly or disappear gradually by the staining method.^1^ Until the 20th century, atherosclerosis and syphilis were recognized as the two most important causes of aneurysm.^2^

In the past 30 years, considerable progress has been made in the research of the pathogenesis of aneurysms under multidisciplinary efforts involving molecular and cellular biology and solid and fluid mechanics.^3^ Throughout a healthy person’s life, the active components of the aortic wall must be constantly regenerated and modified to maintain the integrity and function of the system and to withstand repeated wall stresses. Unfortunately, in some cases, this perfectly stable system becomes destabilized by disease or other complex processes, and part of the aortic wall can permanently weaken and swell, forming an aneurysm, primarily in the abdominal and thoracic portions of the aorta and in the intracranial artery surrounding the circle of Willis. Aneurysms rupture when the dilated wall of an artery cannot support the stress created by the flow of blood inside. Ruptured aneurysms often result in sudden death or severe disability.^3^ The exact cause of the disease is still unknown, but a widely proposed hypothesis has been that specific changes in the hemodynamic forces acting on the aortic walls are a key contributor to the origin and progression of the disease.^4,5^

## Classification

According to where they occur, aortic aneurysms (AAs) are often classified as abdominal aortic aneurysms (AAAs), thoracic aortic aneurysms (TAAs), intracranial aneurysms, and so on. Since smooth muscle cells in the thoracic aorta originate from the neural crest and the somitic mesoderm, whereas smooth muscle cells in the abdominal aorta originate from the splanchnic mesoderm, this distinction leads to a different pathogenesis of TAA versus AAA.^6^

**Fig. 1:**
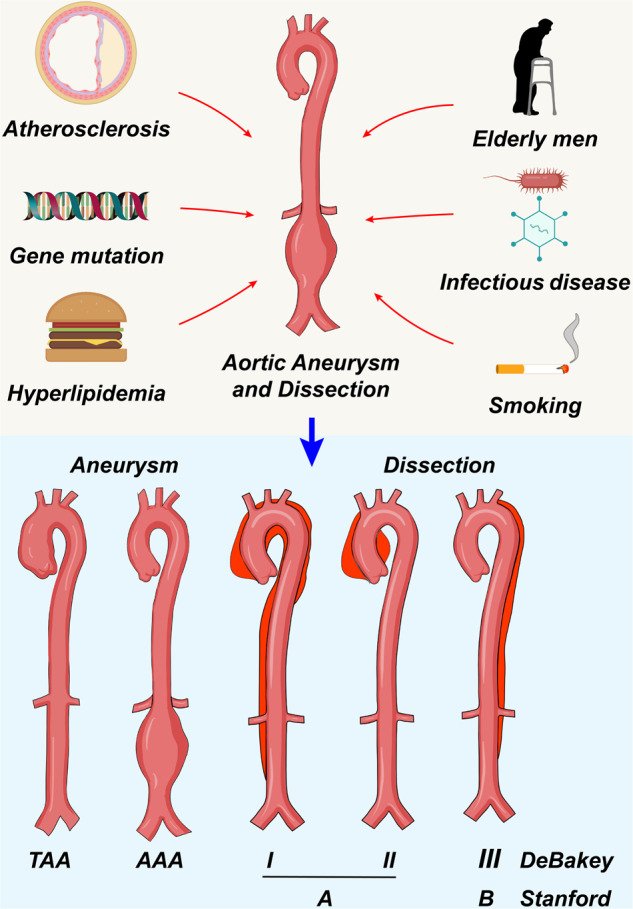
The risk factors for AA formation and the classification of aortic aneurysm and aortic dissection

A dissecting aneurysm, also called aortic dissection (AD), is caused by rupture of the aorta, which flows from the inner hole to the middle layer, causing the wall to be disformed and the blood flow to extend along the longitudinal axis.^7^ Clinical classification of AD and AA are mainly formed on the basis of anatomical location. AD is more likely to occur in the descending aorta with aortic dilatation, which may be because parts of vessels are differentiated from cells of different layers during embryonic development.^8^ According to the De Bakey fractal, AD is divided into the following three types. Type I lesions extend from the ascending aorta to the abdominal aorta, and the lining of the wall is much closer to the ascending aorta. Aortic valve closure is often caused by aortic valve expansion. Type II: the disease is limited to the ascending aorta, which extends to the beginning of the unknown artery. The endometrium is often above the aortic valve, and the aortic valve is not fully closed. Type IIIa: the aorta of the lesion from the lower artery of the left clavicle to the upper lobe of the diaphragm. Type IIIb: lesions from the descending aorta to the abdominal aorta and the iliac artery. According to the Stanford type, it can be divided into two types: Type A: the aortic wall and the ascending aorta are removed; Type B: aortic wall stripping begins with the lower artery opening of the left clavicle, which extends to the descending aorta^9^ (Fig. 1). Because of the heterogeneity in the segmental development of the aorta, the pathogenesis and targeted treatment of AA in different parts of the aorta needs further exploration to achieve better therapeutic results.

## Etiology and risk factors

### Etiology

Most AAAs are nonspecific, meaning there is no known cause.^10^ A few aneurysms have a clear etiology and are secondary to other diseases, such as atherosclerotic disease, trauma, connective tissue disease (Marfan Syndrome, Ehlers‒Danlos Type IV), infectious disease (tuberculosis, syphilis, bacteria, fungi), and inflammatory diseases.^11^

The causal relationship between aneurysm and atherosclerosis has always been controversial. Atherosclerotic diseases and AA have similar risk factors, and atherosclerotic lesions are considered to be the cause of some aneurysms. To address this issue, Dwayne Reed conducted a 20-year follow-up of more than 8000 Japanese men in Hawaii, accumulated clinical and autopsy data, and analyzed the causality of atherosclerosis in the development of AAs. From a preventive perspective, risk factors for aortic atherosclerosis and atherosclerosis itself appear to be essential for causal pathways for the vast majority of AAs in this cohort.^12^ In recent years, an increasing number of studies have suggested that atherosclerotic obstructive disease and AA should be distinguished. Xu et al. evaluated the relationship between atherosclerotic plaque deposition and aortic wall reaction and the development of aneurysmal and occlusive disease of the inferior renal aorta. Morphologic differences at five standardized locations in the infrarenal aorta in 67 male cadaver aortas were studied. The results indicate that there may be different local responses to atherosclerosis in humans. Plaque deposition with local dilation, thinning of the middle membrane, and loss of the medial elastic plate may predispose this aorta to subsequent aneurysm formation. Plaque deposition without thinning of the middle membrane, loss of elastic plates, and dilation of the aortic wall may lead to lumen narrowing of the aorta in the event of continued plaque accumulation.^13^ The vascular response to atherosclerotic lesions is different and may degenerate into dilation or stenosis, with only a small percentage developing into aneurysms.

There could be permanent and abnormal dilation of the aortic wall after trauma to the aorta. CT showed local widening of the aorta with an irregular wall edge. The density of the hematoma on the plain scan was slightly higher than that in the aortic cavity, and the enhancement was relatively low. Different degrees of calcification could be seen between some aortic walls. A portion of blunt abdominal aortic trauma may produce pseudoaneurysm. In one study, 40 children aged 1–16 who experienced blunt abdominal trauma were analyzed. Major aortic injuries included complete aortic wall rupture among 12.5% of the subjects, 70% experienced endometrial transection, and 15% exhibited pseudoaneurysm formation. Symptomatic lesions and complete rupture should be repaired immediately. Peri-circumferential transplantation has a high risk of complications and should also be treated. Some endometrial transection and delayed pseudoaneurysm can be observed by clinical examination and imaging. Patients with the latter pathology should be operated on at any sign of deterioration.^14^

Infectious diseases such as syphilis and tuberculosis and bacterial and fungal infections can cause arteritis in the middle layer, where elastic fibers breakdown and dilate or form localized cystic hematomas. Inflammatory arteritis mainly includes Takayasu’s arteritis, giant cell arteritis and Behcet’s disease. Inflammatory aortitis is the main cause. Aortitis may develop into dilation or even aneurysm, with a high risk of rupture.^15^

Connective tissue diseases include Marfan Syndrome and Ehlers‒Danlos Type IV, and most of them are associated with aneurysm. Marfan syndrome is a systematic connective tissue disorder caused by mutations in the extracellular matrix protein fibrillin 1. Major clinical manifestations include proximal AA, lens dislocation, and long bone overgrowth. Fibrillin 1 is a major component of extracellular matrix microfibers. In patients with Marfan’s syndrome, the aorta demonstrates reduced elastin content and rupture of elastic fibers, resulting in aortic aneurysm or dissection.^16^

### Risk factors

There are many risk factors for AA, including poor lifestyle habits and other chronic diseases, such as smoking, age, high blood pressure, chronic obstructive pulmonary disease, hyperlipidemia, and genetic factors, such as male sex, white race and family history.

Older men are more likely to have AA. Deaths from AA ruptures begin to increase significantly in people over age 65. The prevalence of AAA is six times higher in men than in women, with a 40% increase in risk every five years after age 65.^17^ Studies also found that Blacks, Hispanics, and Asians had a lower risk of AA than Whites and Native Americans.^18,19^

There is a strong clinical correlation between smoking and the occurrence and development of AA. Wilmink T.B.M. studied the exact effect of smoking, duration of smoking, and smoking cessation on the risk of developing AAA and found that smokers were 7.6 times more likely to develop aneurysms than nonsmokers, and former smokers were 3 times more likely to develop aneurysms than nonsmokers.^20^ The duration of smoking was significantly correlated with the risk of aneurysm.^21^ Among all populations, annual smoking increases the risk of rupture of AAA by 4% (95% CI 2%).^20^ According to these clinical observations, long-term smoking may be the most important environmental risk factor for the occurrence and development of AA, but the specific pathophysiological mechanism of smoking affecting the occurrence and development of AA is still not clear. Possible theories include disruption of collagen synthesis, altered expression of matrix metalloproteinases (MMPs), and oxidative stress.^22^

Hypertension is generally considered to be a risk factor for AA, and elevated mean blood pressure is considered to be an independent risk factor for aneurysm rupture, reflecting the ongoing hemodynamic burden on the aortic wall, which leads to wall weakness.^23^ In experimental animal models, hypertension accelerates the progression of experimental aneurysms by upregulating nuclear factor kappa-B (NF-κB) and erythroblast transformation specific (ETS). AAA was produced by elastase perfusion in hypertensive and normal rats. The size of AAA increased rapidly in hypertensive rats compared with normal rats. Western blot analysis showed that the expression of MMP-2, MMP-3, MMP-9, MMP-12 and intercellular adhesion molecules was increased in hypertensive AAA rats, accompanied by upregulation of NF-κB and ETS.^24^ However, the association between hypertension and aneurysm seems to be weak and needs further investigation.^25,26^

Hyperlipidemia is a risk factor for AA, whereas high HDL levels are a protective factor. Iribarren et al. reported that serum cholesterol increases (>240 mg/dl) were associated with an odds ratio (OR) of 2.82 for AAA (95% CI 2.13–3.72).^27^ Pleumeeker et al. reported a protective effect of elevated HDL in plasma on AA.^28^ Diabetes has a protective effect on AA, and Jonathan Golermer found that diabetes is negatively correlated with aneurysms.^29^ Ning et al. studied incident AAA according to baseline glycemic status. Diabetes was independently associated with a lower AAA risk. In addition, with a longer duration of diabetes, the inverse association was more evident.^30^ The possible mechanism is that diabetes mellitus (DM) can also alter the production, degradation and deposition of other glycosaminoglycans in the aorta, with additional consequences on extracellular matrix (ECM) remodeling as well as the structural and physical properties of the aortic wall.^31^ It can be observed that the etiology and risk factors for AA mostly come from other vascular-related diseases. Genetic factors and key pathogenic genes remain to be explored to establish patient pathogenic gene profiles so that AA screening and prevention in high-risk populations can be implemented (Fig. 1).

## Clinical manifestations

### Symptoms

Unruptured aneurysms are usually asymptomatic in most patients and are mostly detected during physical examination, especially in those with coronary, peripheral or cerebrovascular diseases.^32^ Unruptured AAAs may have complications such as distal embolization and acute thrombosis, but acute thrombosis is rare. There are also complications due to pressure on adjacent structures caused by swelling, including lumbar pain when the spine is compressed. Hydronephrosis of the ureter may also occur when the aneurysm is inflammatory or involves iliac bifurcation. Some asymptomatic aneurysms may be detected because of complications.

AAA rupture is a clinical emergency. The patient presents with severe abdominal or chest and back pain that cannot be relieved, accompanied by hypotensive shock, abdominal pulsing mass and other symptoms. This is the typical triad of ruptured AAA, but clinically, typical symptoms are often few, and only 25–50% of patients have this typical triad.^33,34^ The degree of shock varies depending on the location and size of the rupture and when the patient is admitted to the emergency department for evaluation. Rupture of the anterior lateral wall into the peritoneal cavity is more serious and usually causes death. In the event of a rupture of the posterior lateral wall into the retroperitoneal cavity, a small tear can temporarily seal the rupture, and initial bleeding is less likely. However, within a few hours, there will be a larger rupture. This two-phase evolution underscores the importance of the intermediate phase after the initial event, which applies to medical transfers and emergency repairs. Differential diagnosis is needed for acute myocardial infarction, kidney stones, and gastrointestinal diseases such as ulcerative perforation.^35^

### Diagnosis

Most of the patients admitted to the emergency department complain of abdominal or chest and back pain, which requires imaging examination, such as ultrasound or computed tomography (CT), for diagnosis.

Routine abdominal ultrasound imaging is the gold standard for AAA diagnosis and monitoring in asymptomatic patients, and its diagnostic accuracy is close to 100%.^36^ Ultrasound has the advantage of a harmless, noninvasive, affordable portable scanner that can be widely used for screening.

CT is the imaging standard for AAA because it can detect aneurysms complicated by other vessels and provide a basis for the planning of surgical intervention.^37^ CT is also performed with 3D imaging and CT angiography, which provide additional anatomical details, so a CT examination is needed after an aneurysm is found to further determine subsequent therapeutic interventions.

Magnetic resonance imaging (MRI) allows observation of some of the structures adjacent to the aneurysm. MRI combined with magnetic resonance angiography (MRA) enables clear observation of other vessels. MRA uses nonnephrotoxic contrast agents (such as gadolinium). MRA is less harmful than conventional angiography, which uses nephrotoxic contrast agents and has also been used in the further evaluation of aneurysms. In addition to these traditional imaging techniques, functional imaging is also used to assess the pathophysiological pathways involved in aneurysms.

Positron emission tomography (PET) is a clinical imaging method for metabolic and molecular imaging. It uses fluorodeoxyglucose F^18^ (^18^F-FDG) as a tracer to identify areas of increased glycolysis, such as some inflammatory sites and tumors.^38^ Multiple studies have shown that the uptake of ^18^F-FDG in AAA is associated with inflammation and phagocyte infiltration, proteolytic activity of MMPs, and cellular and molecular signal transduction prior to rupture.^39^ However, ^18^F-FDG is nonspecific in the uptake of AAA, and AAA imaging with PET is still a challenge. Nonspecificity of symptoms and imaging dependence of diagnosis challenge the early detection of AA. New molecular targets found in metabolic and immune pathways may help with early screening and process monitoring of AA.

## Mechanism

### Vascular smooth muscle cells

Vascular smooth muscle cells (VSMCs) are the major component of the vessel wall and perform many functions while maintaining the vascular structure. The various changes in VSMCs are an important cause of AA formation.

#### VSMC phenotypic switch

During the formation of the embryonic vascular system, smooth muscle precursor cells are recruited into the vascular network composed of endothelial cells, which are further influenced by various cytokines, such as platelet-derived growth factor-BB (PDGF-BB) and transforming growth factor beta (TGF-β), and then differentiate into mature VSMCs. The ascending and descending aortas are of different origins.^40^ The VSMCs of the ascending aorta develop from the second heart field and the cardiac neural crest, and VSMCs of the descending aorta develop from mesodermal lineages. This difference leads to the distinction between TAA and AAA.^41^

VSMCs are highly plastic and can switch between two phenotypes. The contractile type has a prominent VSMCs phenotype, and the synthetic type has dedifferentiated properties. Contractile VSMCs are spindle-shaped and express high levels of contractile proteins such as α-smooth muscle actin (α-SMA), SM myosin heavy chain (SMMHC), smooth muscle 22α (SM22α), and calponin (CNN).^42^ The contractile phenotype is essential for the aortic wall and maintains aortic strength. In contrast, in the case of inflammation and injury, VSMCs switch to a synthetic phenotype with a strong proliferation and migration capacity, as well as a higher secretion of fibrosis-related proteins and inflammation-related proteins. The expression of osteopontin in the synthetic phenotype is increased, while the expression of contractile proteins is decreased.^43,44^ This process is regarded as a phenotypic switch. In addition to AA, many cardiovascular diseases, such as atherosclerosis and hypertension, are associated with VSMC phenotypic switching.

There are many factors that regulate the phenotypic switching of VSMCs. One of the most well-studied is TGF-β. There are three isoforms of TGF-β, which all have effects on cell differentiation, proliferation, and apoptosis.^45^ Stimulation of VSMCs with TGF-β upregulates the mRNA and protein expression of α-SMA, SMMHC, and CNN while reducing proliferation.^46^ The mainstream view is that TGF-β is a protective factor for AAA. However, it has also been suggested that excessive inhibition of VSMC proliferation may weaken the structure of the aortic wall and lead to AA dilatation.^47^ There is a significant upregulation of TGF-β in individuals with AA.^48^ Therefore, the role of TGF-β is still controversial.

There are two common TGF-β pathways, both of which play an important role in AA. TGF-β has two receptors, TGF-β receptor (TGFBR)-1 and -2, which are downstream of decapentaplegic protein (SMAD)-dependent or non-SMAD-dependent pathways after binding to TGF-β. SMAD2 or SMAD3 is activated and phosphorylated by the TGF-β-TGF-βR complex, leading to SMAD4 nuclear ectopic, which in turn affects the transcription of downstream contractile proteins.^49^ In the non-SMAD-dependent pathway, TGF-β initiates the Ras Homolog Family Member A (RhoA) and mitogen-activated protein kinase (MAPK) cascades, the latter including extracellular signal-regulated kinase (ERK), Jun N-terminal kinase (JNK) and p38. TGF-β activates and phosphorylates them separately, which in turn affects the transcription of downstream contractile proteins.^50,51^ It has also been shown that TGF-β inhibits vascular chronic inflammation and attenuates AAA development by suppressing the activity of inflammation-related signaling pathways such as signal transducer and activator of transcription 3 (STAT3) and NF-κB.^52^

In Marfan’s syndrome, which is frequently associated with the development of AA, mutations in fibronectin 1 (FBN1) occur. FBN1 restricts TGF-β signaling. Hence, it is suggested that mutated FBN1 fails to bind to TGF-β, leading to TGF-β overexpression, which in turn promotes the development of AA.^53^ However, a different view has been proposed because in the mouse model of Marfan’s syndrome, an increase in TGF-β levels in the aorta was not found. Instead, physiological SMC TGF-β signaling protects against the AA associated with Marfan’s syndrome.^54^ Related studies are continuing. In addition, in Loeys‒Dietz syndrome with TGF-β receptor mutations, AA or AD has also been observed.^55,56^

Because of the important role of TGF-β, its related treatment is also under investigation. It has been shown that the neutralizing therapy of TGF-β (i.e., exogenous injection of TGF-β antibodies) can aggravate the progression of AA and promote AA rupture.^57^ It has also been demonstrated that specific blockade of TGF-β in VSMCs causes aortic wall thinning.^58^ Growth differentiation factor 11 (GDF11), a member of the TGF-β,^59^ cyclophilin A^60^ and vitamin B^61^ groups, can affect the formation and progression of AA through TGF-β-related pathways.

miRNAs are another common factor affecting VSMC phenotypic switching. Micro-RNAs are RNA strands of 19–24 nucleotides that regulate gene expression and function to repress gene expression by degrading messenger RNAs or mimicking small interfering RNAs (siRNAs) to inhibit translation. The decrease in miR-23b in AA leads to the upregulation of the transcription factor forkhead box O4, which in turn promotes VSMC switching to the synthetic phenotype and thus exacerbates the disease.^62^ The miR-143/145 cluster could inhibit the transcription of proteins such as Kruppel-like factor 4 (KLF4) and ETS Like-1 protein, which in turn switches VSMCs to a contractile phenotype.^63,64^

Other factors include mitochondrial protein and KLF4, all of which can affect the development of AA by regulating the phenotypic switch of VSMCs.^65,66^ However, the effects of all proteins are compounded and may affect VSMC phenotypic switching along with apoptosis and degradation of the ECM. Therefore, all the factors should be considered comprehensively.

#### VSMC death

In AA, a decrease in the number of VSMCs can be observed, which could further lead to a decrease in the ECM and weakening of the aortic wall.^67^ VSMCs are mainly apoptotic in AA. With research progress, other types of cell death, such as ferroptosis and pyroptosis, have been gradually identified.

The apoptosis of VSMCs can be found in the aortas of AA patients and AA model mice.^68^ Multiple factors can promote apoptosis of VSMCs. When macrophages infiltrate the aortic wall during AA, they express a large number of inflammatory factors, such as interleukin 6 (IL-6), tumor necrosis factor-α (TNF-α), and monocyte chemoattractant protein-1 (MCP-1). Many of them promote VSMC apoptosis.^69^ Biomacromolecules such as oxidized low-density lipoprotein could also promote apoptosis. The TGF-β could inhibit apoptosis and play an AA protective role. In addition, endoplasmic reticulum (ER) stress occurs in VSMCs due to cytokine stimulation and mechanical stretch stimulation, which in turn cause programmed cell death.^70,71^ Therefore, targeting the apoptosis of VSMCs is one of the strategies to treat AA. Transcription factor EB (TFEB) can inhibit apoptosis but is downregulated in the tissues of AA patients. 2-Hydroxypropyl-β-cyclodextrin (HPβCD) can activate TFEB and inhibit AA in a mouse model and is a potential clinical therapeutic agent.^72^

Pyroptosis is an acute cell death mediated by the caspase family. Exogenous factors lead to the formation of inflammasomes, which in turn lead to the formation of pores on the cell membrane by the gasdermin (GSDM) family. Finally, cytokines such as IL-1β and IL-18 are released.^73,74^ In human and mouse AA tissues, the NOD-like receptor thermal protein domain-associated protein 3 (NLRP3)-Caspase-1 system is activated, indicating that pyroptosis may occur in VSMCs and Nlrp3-deficient mice exhibit a lower incidence of AA.^75,76^ However, the pyroptosis pathway is not the only pathway downstream of the inflammasome, and supporting evidence of VSMC-specific knockout animals is lacking. Therefore, the role of VSMCs in pyroptosis remains to be investigated.

Ferroptosis is a kind of programmed cell death dependent on iron. System Xc^−^ and glutathione peroxidase 4 (GPX4) inhibition are the main causes of ferroptosis. System Xc^−^ is a cystine/glutamate antiporter system that transfers glutamate out the cell and imports cystine to participate in the generation of glutathione (GSH).^77,78^ In addition, abnormal transferrin and iron regulatory protein 2 (IRP2) increase intracellular iron and disrupt iron homeostasis, which directly leads to reactive oxygen species (ROS) production.^79^ Ultimately, abnormal ROS accumulation leads to death accompanied by inactivation of essential enzymes and DNA damage.^80^ There are few studies related to ferroptosis in AA. It has been reported that the iron level, ferroptosis-related molecules transferrin receptor (TFR), heme oxygenase 1 (HOMX1), ferritin and the lipid peroxidation product 4-hydroxynonenal are increased in AD. The histone methyltransferase inhibitor BRD4770 has a protective effect against ferroptosis in VSMCs, which in turn attenuates AD in mice.^81^ Cigarette smoke extract could trigger AA by inducing ferroptosis in VSMCs and upregulating other cytokines, such as IL-6, MMP-2/9, and TNF-α, suggesting that VSMCs may promote the development of AA through ferroptosis.^82^

#### ECM changes

VSMCs are involved in the synthesis and processing of ECM. The ECM contains proteoglycans, glycoproteins, laminin, collagen, and elastic fibers that maintain the strength and elasticity of the vessel wall.^83,84^ In AA, ECM degrades and leads to the fragmentation and dilatation of the vessel wall.^85^ One of the main proteins involved in degradation is MMPs, and other proteases, such as a disintegrin and metalloproteinases (ADAMs), are also involved in this process.

Among the MMP family, MMP-2/9 is the most studied matrix metalloproteinase. High levels of MMP-2/9 expression can be observed in AA tissue. MMP-2 and MMP-9 are barely expressed in normal aortic tissue and are more highly expressed in early AAA than in late or ruptured AAA, suggesting that MMP-2 may be more critical for the early formation of AA.^86^ With inflammatory stimulation, the content of MMP-9 in aortic tissue is upregulated.^87^ Additionally, as the content of MMP-9 in aortic tissue increases, the content of protein hydrolases is also upregulated, which exacerbates the risk of AA rupture.^88^

The activity of MMPs is regulated by tissue inhibitor of matrix metalloproteinases (TIMPs). Four members of the TIMP family (TIMPs-1/2/3/4) can inhibit all MMPs and a large number of ADAMs through a covalent bond between their N-terminus and protease catalytic domains.^89^ AA has been observed in TIMP1 knockout mice^90^ and a significant decrease in TIMP-2 in the aortic wall of AAA patients.^91^ In a mouse model, aneurysms in TIMP-3 knockout mice were more prone to rupture, leading to death.^92^

#### VSMCs autophagy

Autophagy is a lysosome-mediated process that removes damaged proteins and organelles. Autophagy is regulated by highly conserved autophagy-related genes (ATGs). Impaired autophagy may lead to ER stress, ROS damage and cell death. Autophagy plays an important role in the physiological processes of VSMCs associated with aging and atherosclerosis.^93^ Autophagy has been found to be associated with VSMC phenotypic switching.^94^ In the aortic tissue of TAA patients, lower levels of autophagy could be observed.^95^ After VSMC-specific knockdown of the autophagy-related gene Atg5, an increased incidence of AA in mice with increased ER stress levels and upregulated inflammation was observed, and these phenomena were also verified in human AA tissues.^96^

The above section describes the role and mechanism of VSMCs in AA, such as phenotype switching, cell death, autophagy, and ECM regulation. As an important component of the aorta, VSMCs could receive more attention as a target for AA prevention and treatment.

### Endothelial cells

Endothelial cells (ECs) are in the inner layer of the aorta and play a major role in maintaining aortic homeostasis. An increasing number of studies have shown that endothelial cell dysfunction is an important factor for AA.

#### Oxidative stress

In response to external stimulation, such as altered blood flow and inflammatory factors, ECs may undergo oxidative stress, which in turn leads to the accumulation of ROS. Endothelial nitric oxide synthases (eNOS) can produce nitric oxide (NO); in turn, NO promotes vasodilation. In addition to endothelial NOS (NOS3), there are also neuronal-derived NOS (nNOS, also called NOS1) and inducible NOS (iNOS, also called NOS2).^97^ However, in the absence of tetrahydrobiopterin (HB4) and the key enzymes GTP cyclohydroxylase 1 (GCH1) and dihydrofolate reductase (DHFR), uncoupling occurs, and superoxide O_2_^−^ is produced.^98,99^ O_2_^−^ has been found to be significantly increased in human AAA tissue. O_2_^-^exacerbates vascular oxidative stress levels, causing a remodeling of the ECM and apoptosis of VSMCs, which leads to exacerbation of AA.^100^ It has also been noted that O_2_^–^ produced by nicotinamide adenine dinucleotide phosphate (NADPH) oxidase can interact with NO to generate ONOOO^-^, an ion with strong oxidative properties that can cause further uncoupling of eNOS, which in turn leads to more severe oxidative stress.^101^ Adding HB4 or HB4-producing folic acid resulted in the remission of AA in a Marfan syndrome mouse model.^102^

In addition to eNOS, iNOS may also cause superoxide production in aortic tissue. When ROS are present, they induce upregulation of iNOS levels and produce excess NO to bind to superoxide O_2_^-^; the resulting ONOOO^-^ amplifies the oxidative stress effect.^101^ It has also been found that excessive NO could be produced after activation of iNOS and may promote atherogenesis.^103^

Another important source of ROS in ECs is NADPH oxidases (NOXs). NOX is a complex containing several regulatory subunits, e.g., p40phox, p47phox, p67phox, rac1 and cytochrome b558 (containing a catalytic Nox subunit and a p22phox subunit). ECs mainly express NOX2 and NOX4. In stimulations with angiotensin (Ang) II and high glucose, endothelial NOX2 is activated, and ROS are generated, leading to endothelial dysfunction and vascular injury.^104^ It has been reported that mice overexpressing NOX2 produce more severe AD. ECs overexpressing NOX2 could increase ERK1/2 phosphorylation in VSMCs by secreting cyclophilin A (CypA), which in turn upregulates oxidative stress and inflammation in VSMCs.^105^ By using inhibitors to block the phosphorylated p47phox interaction with p22phox in NOX2, it is possible to attenuate AA progression in mice by inhibiting oxidative stress.^106^

#### Biomechanical stress

Biomechanical stress is an important factor affecting vascular homeostasis. Blood flow exerts a variety of forces on the aortic wall, including circumferential and longitudinal stresses as well as shear stress, which is divided into unidirectional laminar shear stress (LSS) and oscillatory shear stress (OSS). ECs are very sensitive to biomechanical stress and are stimulated in areas of stenosis and branching, where wall shear stress increases.^107,108^ This is why aneurysms often occur in the region of the aortic arch and infrarenal aorta.^109^ Shear stress activates the PKC pathway,^110^ JNK pathway,^111^ etc., in ECs through the mechanosensory complex platelet and endothelial cell adhesion molecule 1 (PECAM-1) on the cell membrane surface.^112^ The downstream pathways are activated, and one of the important effects is the level of eNOS. Shear stress promotes the synthesis of eNOS in ECs through factors such as scavenger receptor class B type 1 (SR-B1)^113^ and c-Src-tyrosine kinase.^114^ Then, eNOS promotes the production of NO. NO promotes EC diastole, which in turn reduces the damage caused by shear stress to the vessel wall. In mice with eNOS knockout, an exacerbation of AA was observed.^115^

Blood flow tension could also alter the protease expression and activity of ECs. OSS can promote MMP activity in the aortic wall while decreasing the level of TIMP-3.^116,117^ Additionally, high expression of cathepsin was detected in ECs of AA patients, suggesting that ECs can promote the progression of AA by degrading ECM.^118^ In addition, vascular endothelial growth factor (VEGF) and cell adhesion molecules vascular cell adhesion molecule 1 (VCAM-1) and intercellular adhesion molecule 1 (ICAM-1) have been found to be upregulated in high shear stress, leading to gaps between ECs while increasing the adhesion of inflammatory cells to ECs and VSMCs.^113,119,120^

#### Intraluminal thrombus

Almost 75% of AAAs have intraluminal thrombus (ILT) formation,^121^ which has been mentioned in several articles and reviews.^122–124^ ILTs are usually accompanied by destruction of the adjacent endothelium and contain a large number of macrophages, neutrophils, erythrocytes and platelets, as well as a large number of proteases, such as MMPs.^125–127^ There are two opposing views on the role of ILT in AA in recent years. Some researchers believe that ILT creates a harmful environment that affects oxygen transport in the aortic wall and aggravates inflammation.^128,129^ However, some articles have also found that ILT may decrease aortic wall stress and play a buffering role from a biomechanical perspective.^130–132^ Researchers are now considering that both conditions occur simultaneously in ILT. Even though ILT alleviates aortic wall stress, insufficient oxygen transport and increased levels of inflammation produce thinning and degradation of the aortic wall in this region, which in turn promotes AA rupture. Overall, the effect of ILT on AA remains pathological rather than protective.^127,133^ Therefore, anticoagulant treatment could reduce ILT assessment and demonstration, but whether it can reduce the possibility of AAA rupture requires further study.^134^ Because of the property of ILT to promote AA rupture, several studies have shown that ILT measurement by CT or three-dimensional contrast-enhanced ultrasound can predict the risk of AAA rupture and could be used for clinical purposes.^135^ Similarly, data about AA, such as ILT, demonstrate that vessel radius and blood flow rate can be mined by deep learning and artificial intelligence techniques for AAA growth prediction, which can assist in diagnosis and treatment in the clinic.^136^

### Immune cells and inflammatory factors

The immune response plays an important role in the course of AA. Immune cells not only secrete inflammatory factors that lead to apoptosis of aortic wall cells and VSMC phenotype switching but also secrete proteases that induce ECM degradation and lead to aortic dilation and rupture. In AA tissue, neutrophils, macrophages, natural killer (NK) and natural killer T (NKT) cells, T and B cells are infiltrated.^137,138^ Together, these cells contribute to the level of inflammation in the aorta from multiple perspectives.

#### Adaptive immunity

There is a large infiltration of immune cells in AA tissue, including T cells with B cells. T cells are mainly CD4 + T cells, which are the most abundant immune cells infiltrating AA.^139^ When CD4 + T cells are not present, the development of AA is significantly inhibited.^140^ Under different stimuli, CD4 + T cells can differentiate into different T cells, such as Th1 cells, Th2 cells, Th17 cells, regulatory T (Treg) cells and T follicular helper (Tfh) cells.^141^ All these T cells are involved in the formation of AA, and increased levels of TH1, TH17 and TH22 have been reported in vascular tissues of AA patients.^142^ There is an upregulation of Th17-, Th1-, Th9-, Th22- and Tfh-specific (TF) cells in the blood of AA patients and a downregulation of Th2 cells and Treg cells.^143,144^

The differentiation of TH1 cells depends on the stimulation of IL-12, which in turn secretes interferon-γ (IFN-γ) to promote the activation and recruitment of macrophages. Inhibition of IL-12 levels with antibodies could inhibit macrophages and thus AAA progression.^145^ TH17 cells are induced by stimulation of IL-23, IL-1, and IL-6 and promote macrophage activity by secreting IL-17.^146^ After knockdown of IL-17 in mice, the progression of AA was inhibited.^147^ Additionally, inhibition of AA was observed after inhibition of the pathway that activates TH17 cell differentiation.^148^

Th2 cells are considered anti-inflammatory in the current view. IL-4 stimulates their differentiation. Th2 cells secrete IL-4, IL-5, IL-10 and IL-13 through the STAT6 and GATA-3 pathways. IL-4 and IL-10 limit the cytotoxic potential of macrophages and reduce the expression of MMPs.^149^ Treg cells are specific CD4 + T cells that regulate other T-cell subsets and thus inhibit proinflammatory effects.^150^ Both IL-2 and TGF-β can stimulate Treg cells, which in turn increases the secretion of IL-10 and TGF-β, suppressing macrophage inflammation levels and clearing immune T cells.^151^ The process may also inhibit VSMC apoptosis by secreting trefoil factor 1 (TFF1), which in turn inhibits disease progression in AA.^152^ Downregulation of Treg cell levels was observed in both AA patients and AA mouse models.^153,154^

B cells play an important role in adaptive immunity by secreting antibodies. B cells mainly include B1, B2 and regulatory B cells.^155^ Although B cells account for only 4% to 5% of infiltrating cells in the AA vascular wall, B cells still play an important role. In mice lacking B cells and in mice antagonizing B cells with rituximab mimetic, reduced aortic immune cell infiltration and inflammation were observed, along with a reduction in AA symptoms.^156,157^ B2 cells are the predominant subtype of B cells. B-cell-activating factor (BAFF) can activate B-cell differentiation into the B2 type, and the use of BAFF antagonists can protect mice from AA disease.^158^ Since B cells mainly secrete antibodies, accumulation of these immunoglobulins in mouse AA tissues may induce secretion of IL-6 and MMP-9, damage the aortic wall, and aggravate AA disease.^159^ It has also been reported that anti-β2GPI IgG secreted by B cells may promote AA by causing hyperhomocysteinaemia.^160^

#### Neutrophils

Neutrophils are the most abundant cells in the human immune system and are the immune cells that respond fastest.^161^ In traditional studies, neutrophils accomplish the immune response through phagocytosis and degranulation. Neutrophils contain a large number of antimicrobial proteins, such as lysozyme, lactoferrin and cathepsin. These proteins are released in the pathogen or directly outside the cell after phagocytosis in neutrophils. Neutrophils are the main source of MMPs in AA.^162^ Neutrophil elastase (NE) released from neutrophils can exacerbate AAA by activating MMPs and inactivating its inhibitor TIMP. The use of neutrophil antibodies to induce neutropenia can effectively suppress AAA.^163^ In an elastase-induced AAA mouse model, ECs release the family with sequence similarity 3, member D (FAM3D), which promotes the recruitment of peripheral blood neutrophils to the abdominal aorta by activating the downstream protein kinase C (PKC), ERK, and p38MAPK pathways through the activation of membrane attack complex-1 (Mac-1) and formyl peptide receptor 2 (FPR2) in neutrophils, thereby inducing the development of AAA.^164^

A novel type of neutrophil killing called neutrophil extracellular traps (NETs) has been identified in the past two decades. NETs are neutrophils that enter a death program after stimulation by factors such as IL-1β, where the nuclear membrane ruptures and the nuclear contents are released into the cytoplasm. Finally, the plasma membrane ruptures, and the granule protein-modified chromatin is released outside the cell.^165^ These complexes contain DNA and histones with a variety of proteases, such as NE, cathepsin G, and myeloperoxidase. The involvement of NETs is crucial in the formation of AA, which can aggravate the development of AAA by degrading and weakening the vascular wall. In addition, NETs can activate NLRP3 in macrophages, which further release IL-1β and IL-18 and upregulate inflammation levels in the aorta.^166,167^ Citrullinated histone H3, a marker of NETs, is significantly upregulated in the plasma and arterial tissue of patients with AA and decreases after surgical repair of AA.^168^ NETs are formed in the first 2–3 days of AA formation in mice. Inhibition of NET production by drugs or promotion of NET degradation can effectively inhibit AAA development.^169,170^ It has also been shown that oxidized low-density lipoprotein (oxLDL) promotes the release of NETs, which in turn exacerbates AAA, while high-density lipoprotein (HDL) has little effect on the release of NETs.^171^

#### Macrophages

Circulating macrophages are the main source of macrophages in aortic tissue, and a small number of macrophages reside in the tissue during development.^172–174^ When local vascular tissue is stimulated, chemokines and inflammatory factors are released, and monocytes in the circulating blood are recruited into the vessel wall. When there are more monocytes in the circulating blood, the chance of AA development rises.^175^ Traditionally, monocytes are thought to be differentiated into two types of macrophages, M1 or M2, both of which are upregulated in AA tissues.^176,177^ M1 cells are known as proinflammatory macrophages and can be activated by cytokines such as lipopolysaccharide (LPS) or IFN-γ, which in turn secrete cytokines such as IL-6, TNF-α, MCP-1 that upregulate inflammation, as well as MMPs that breakdown ECM and weaken the aortic wall. M2-type macrophages are called anti-inflammatory macrophages. They can secrete TGF-β or IL-10 to exert anti-inflammatory effects and can repair the ECM.^178,179^ A high M1/M2 ratio can be observed in the aortas of AAA mice.^180^ Notably, Boytard et al. found lower levels of M2 cells and higher levels of M1 cells in the extravascular membrane at the AA site, but the opposite was found in the study of Dutertre et al.^181,182^. This may occur because M1- and M2-type cells are constantly transformed over the course of the disease. It is currently believed that in the early phase of AA, the M1/M2 ratio is high, which in turn produces vascular destruction. In the later phase of the disease, with hemodynamic changes and secretion of factors such as miRNA, macrophages polarize toward M2 to repair the vessels.^183,184^ This also reflects the phenomenon of interconversion of two roles of macrophages to maintain tissue homeostasis in the disease.^174^ In our laboratory, we have discovered that macrophages upregulate circulating levels of succinate, which is generated through the p38α-cAMP-response element-binding protein (CREB)-oxoglutarate dehydrogenase (OGDH) axis. Thereby, ROS were promoted, and the macrophages were converted to M1, which prompted AAD progression.^185^

#### Cytokines

Cytokines play an important role in AA. Cytokines are mainly secreted by immune cells and affect the level of inflammation in vascular tissue and the degradation of ECM. In a mouse model of AA, cytokines change significantly.^186^ We review several cytokines that are important in AA disease.

##### Interleukin (IL)-6

IL-6 is an important inflammatory factor involved in many proinflammatory processes in the body.^187^ IL-6 levels are significantly increased in vascular tissue as well as in circulating blood in AA patients, and circulating blood IL-6 levels may be proportional to the severity of the aneurysm.^188–190^ Animal experiments have demonstrated that IL-6 concentrations in vascular tissues in Ang-II-induced AA mice increase over time.^191^ The development of AA disease in mice could be inhibited after administration of monoclonal antibodies against the IL-6 receptor.^192^ Mechanistically, there are two signaling pathways for IL-6, namely, classical and trans-signaling. Classical signaling refers to IL-6 binding to the IL-6 receptor (IL-6R) on cells, and trans-signaling refers to IL-6 activation after binding to free soluble IL-6R, followed by binding to gp130 on a variety of cells.^193^ Subsequent activation of the STAT3 pathway promotes the differentiation of monocytes into macrophages, overactivation of VSMCs, and recruitment of macrophages, among other pathological changes in AA.^194,195^ The use of bazedoxifene can inhibit the IL-6/GP130/STAT3 signaling pathway and thus the progression of AA.^196^ However, it has also been shown that prophylactic IL-6 inhibition has little effect on the formation of AA.^197^

##### Interleukin (IL)-1β

IL-1β is an important factor in the regulation of infectious and aseptic inflammatory responses and is mainly produced by macrophages. Pro-IL-1β, the IL-1β precursor, is not biologically active but requires cleavage to IL-1β by caspase-1 to be active.^198^ Elevated levels of IL-1β have been observed in both the tissue and plasma of patients with AA disease.^199,200^ In mouse models, knockdown of IL-1β with IL-1R inhibits AA formation.^201^ In terms of therapeutic prospects, both the downregulation of IL-1β by antibodies and the inhibition of IL-1R with the antagonist anakinra inhibit the progression of AA.^199,202^ IL-1β plays a central role in mediating the inflammatory response and mediates the activation of secondary inflammatory factors such as IL-6.^203^ Mechanistically, IL-1β promotes the upregulation of EC adhesion factors, thereby recruiting immune cells.^204,205^ Meanwhile, IL-1β can degrade ECM and weaken aortic wall strength by promoting MMP-2/9 expression.^206^ Mechanistically, IL-1β stimulates the phosphorylation of the SMAD4 pathway in VSMCs, which in turn regulates the phenotypic switch of VSMCs.^207^ Meanwhile, IL-1β activates the NF-κB pathway and increases the levels of MMP-2/9 and IL-6.^208^

##### Tumor necrosis factor-α (TNF-α)

TNF-α is a potent proinflammatory cytokine that has been shown to be upregulated in patients with AA.^209^ A study of single nucleotide polymorphisms (SNPs) in healthy populations and AAA patients found that the TNF-α-238G/A gene was associated with an increased risk of AA development.^210^ In elastase-induced AA mice, the number of TNF-α-secreting macrophages was significantly increased.^145^ Animal experiments have demonstrated that TNF-α promotes the inflammatory response during aneurysm progression. Mechanistically, TNF-α deficiency inhibits macrophage polarization toward the M1 type. TNF-α deficiency also attenuates MMP-2 and MMP-9 expression by blocking macrophage activation.^211,212^ In AAA patients and in Ang-II-induced AA mice, TNF-α upregulated galectin-1 (Gal-1) in VSMCs and fibroblasts and subsequently induced MMP-9 expression.^213^ The use of klistatin inhibited TNF-α-induced oxidative stress and apoptosis, which in turn inhibited the development of AAA.^214,215^

##### Monocyte chemoattractant protein-1 (MCP-1)

MCP-1, also known as C-C motif chemokine ligand 2 (CCL2), is an important chemokine that rapidly promotes the adhesion of migrating macrophages to E-selectin expressed by ECs.^216^ In both mouse models and patients with AA, the expression of Mcp-1 in aneurysmal tissue was significantly elevated.^217^ Elevated levels of Mcp-1 in serum exacerbate AA in the atherosclerosis mouse model, and the process can be reversed by knockdown of its receptor C-C chemokine receptor type 2 (CCR2).^218^ In vitro experiments showed that Mcp-1 could upregulate the expression of Fas ligand (FasL) on the surface of macrophages, promote the cytotoxic effect of macrophages and cause autophagy in VSMCs.^219^ In VSMCs, Mcp-1 can induce the expression and activity of MMP-9 by activating the ERK1/2, p38MAPK pathway. It was found that hypoxia upregulates MCP-1 in VSMCs, in addition to causing macrophage chemotaxis and upregulating IL-6 expression in THP-1 cells, which in turn leads to apoptosis via STAT1 in VSMCs.^220^ On the other hand, Mcp-1 can also promote aneurysm repair.^221^ Slow-release Mcp-1 promoted carotid aneurysm growth and healing in mice in a dose-dependent manner. In this process, Mcp-1 upregulates the expression of inflammatory proteins such as macrophage inflammatory protein-1a/2 (MIP-1α, MIP-2) in macrophages and promotes the migration and aggregation of fibroblasts, macrophages, ECs, and VSMCs. Blocking MIP inhibits the repair function of Mcp-1, and the protein-coated cycle of Mcp-1 has been suggested as a possible treatment for AA.^222^

##### NOD-like receptor thermal protein domain-associated protein 3 (NLRP3)

NLRP3 is an innate immunity receptor that recognizes multiple stimuli from pathogens and injured or dead cells and is involved in the pathogenesis of aseptic inflammatory diseases.^223^ In response to stimuli such as pathogen-associated molecular patterns (PAMPs) or damage-associated molecular patterns (DAMPs), NLRP3 activates and leads to the assembly of the NLRP3 inflammasome, resulting in the activation of caspase-1, which in turn causes the secretion of IL-1β and IL-18.^224^ Activated caspase-1 also induces an inflammatory cell death process called pyroptosis by cleaving gasdermin D (GSDMD). The NLRP3 inflammasome consists of the sensor NLRP3, the adaptor apoptosis-associated speck-like protein containing a caspase-recruitment domain (ASC), and the effector enzyme pro-caspase-1. NLRP3 is highly expressed in innate immune cells such as macrophages and neutrophils but also in nonimmune cells such as ECs, cardiomyocytes, fibroblasts, and epithelial cells, which in turn affect the pathological progression of cardiovascular diseases, including atherosclerosis, aneurysms, and vascular injury.^225,226^

Levels of NLRP3 inflammasome effector mediators are elevated in both clinical AAA patients and AAA animal models. As mentioned previously, IL-1β levels were elevated in the plasma of AAA patients. The mRNA and protein levels of IL-18 were also increased in the aorta of AAA patients compared with nonaneurysmal controls.^227^ In aneurysmal tissue, an earlier study by Schonbeck et al. showed elevated levels of caspase-1 in AAA patients. AA patients had elevated levels of NLRP3 mRNA in the aorta.^228^ It was also shown that high levels of plasma IL-1β were observed in individuals who were homozygous for the common C allele of NLRP3 rs35829419, suggesting that genovariation of the NLRP3 inflammasome may play an important role in the progression of AAA.^229,230^ Furthermore, direct knockdown of the NLRP3 inflammasome component was also able to significantly reduce the incidence and severity of AAA while decreasing the inflammatory response, including IL-1β secretion, MMP activation, and elastic lamina degradation.^76^

Recently, it was found that the NLRP3 inflammasome can also affect AAA through other pathways in addition to involvement in inflammatory factor secretion. Activated caspase-1 is able to directly bind and cleave contractile proteins of VSMCs in the aortic wall, such as tropomyosin and myosin heavy chain. Knockdown of NLRP3 and caspase-1 significantly reduced the degradation of these proteins and attenuated the formation of AA and AD in the Ang-II model fed a high-fat diet.^75^ In macrophages, caspase-1 activates MMP-9 by directly cleaving its n-terminal inhibitory domain. In wild-type mice fed a high-fat and high-cholesterol diet, treatment with the NLRP3 inhibitor MCC950 prevented aortic dilatation and dissection in different segments of the thoracic and abdominal aorta.^231^

### Reactive oxygen species (ROS)

ROS and oxidative stress play an important role in the formation of AA, and a rise in oxidative stress has been observed in both patients and mice.^232^ ROS include small reactive ions and molecules such as superoxide (O_2_^-^) and hydrogen peroxide (H_2_O_2_). Low levels of ROS act as signaling molecules and are essential for maintaining normal vascular function. Uncontrolled overproduction of ROS exacerbates oxidative stress, leading to vascular cell injury, such as proliferation and migration of VSMCs, recruitment of inflammatory cells and activation of MMPs.^233,234^ In addition to the role in ECs described above, ROS promote the progression of AA in both macrophages and VSMCs. It has been reported that normal levels of H_2_O_2_ generated via NOX4 promote the differentiation of embryonic stem cells into VSMCs.^235^ In addition, in VSMC-specific overexpression of catalase in AA mouse models, a reduction in VSMC apoptosis and an attenuation of AA could be observed.^236^

The production of ROS is dependent on NOX, uncoupled eNOS (also known as NOS3), mitochondria and xanthine oxidase (XO).^237^ The main function of NOX is to produce ROS, so NOX is the most critical enzyme affecting ROS in AA.^238^ We have mentioned the role of NOX2 and NOX4 above. NOX1 and NOX2 only produce O_2_^-^, NOX4 only produces N_2_O_2_, while NOX5 produces both products.^239^ p22phox, p47phox, NOX2, NOX5 and p22phox expression levels are increased in AA patients.^100,101^ AA was suppressed after NOX1 knockdown or application of the NOX inhibitor apocynin, accompanied by a decrease in MMP-2/9 levels.^240,241^ Superoxide production and eNOS uncoupling activity were significantly reduced in NOX mutant mice and thus caused the suppression of AA.^242^

In addition to NOX, MPO is also involved in the production of ROS in AA. MPO causes H_2_O_2_ to react with Cl^-^ to form HOCl^-^, an ion that reacts with various biomolecules, such as proteins, lipids, and nucleic acids, and causes oxidative damage.^243^ Since neutrophils are involved in the early infiltration of AA formation and neutrophils are the main source of myeloperoxidase production in the body, myeloperoxidase also plays an early role in influencing the level of oxidative stress.^163^ Both the knockdown of MPO and supplementation with taurine to inhibit MPO activity inhibit AA formation.^244^

The antioxidant system also plays a key role. In circulating neutrophils of AA patients, lower levels of catalase are detected.^245^ Tamoxifen inhibits AA formation by upregulating the level of catalase in the aorta.^246^ Superoxide dismutase (SOD) also scavenges ROS. Different results have been reported in tissues from AA patients, with both increases and decreases.^247,248^ This may be due to the different selection of control samples. An increase in SOD mRNA levels in the aorta of AA-molded rats has been observed in animal models.^249^

ROS are also of interest as therapeutic targets for AA. The administration of the antioxidant vitamin E reverses the formation of AA.^250^ Other natural antioxidants, such as polyphenols/flavonoids, have been shown to have antioxidant mitigating effects in cardiovascular diseases, and their effectiveness in treating AA is promising.^251,252^ In addition, drugs such as statins and irbesartan have also shown effects in scavenging ROS, and clinical studies are continuing.^253,254^

**Fig. 2:**
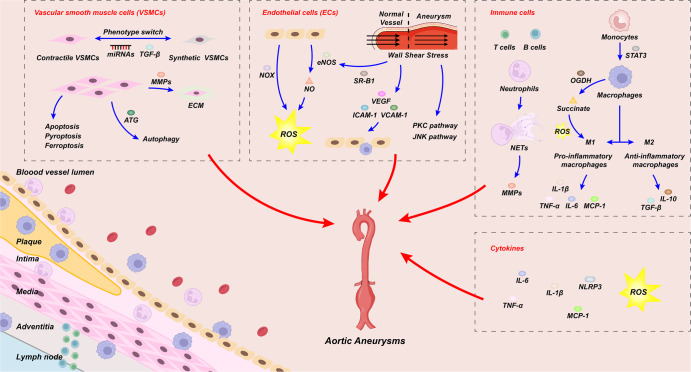
Potential mechanisms of AA

In this section, we review the possible mechanisms underlying the pathogenesis of AA. These include the VSMC phenotype switch, apoptosis, autophagy and effects on ECM; oxidative stress; the role of shear stress and ILT formation in endothelial cells; the release of various cytokines, such as IL-1/6, brought by immune cells; the release of granzyme and the formation of NETs in neutrophils; the proinflammatory phenotype switch of macrophages leading to increased levels of inflammation; and the involvement of ROS leading to aortic cell injury (Fig. 2). As research on AA mechanisms continues, we hope to examine different cells, such as fibroblasts, on AA and find more pathways as potential targets for AA therapy.

## Clinical cohort study

Multiple clinical cohort studies of aneurysms help us understand the natural history of aneurysms and their relationships, such as sex, age, aneurysm size, aneurysm location, and risk of aneurysm rupture. We briefly summarize some of the information related to the clinical cohort of patients with aneurysms (Table 1).

**Table 1:** Summary of aneurysm-related clinical cohort

Author(s) and year of publication	Number of cases	Data sources	Research content
Greving et al.^255^	8382	Six studies from previous clinical cohorts	The correlation between age, hypertension, history of subarachnoid hemorrhage, aneurysm size, aneurysm location and geographic area and aneurysm rupture was analyzed.
Korja et al.^257^	118	Helsinki University Central Hospital	For the first time, the natural life-long course of unruptured intracranial aneurysms (UIAs) was determined and patients at high and low risk of rupture were identified.
Mocco et al.^260^	255	International Study of Unruptured Intracranial Aneurysms database	Factors affecting the rupture risk of unruptured intracranial aneurysms were identified by studying morphological characteristics such as vertical height and size ratio of aneurysms.
Goertz et al.^267^	252	University Hospital of Cologne	To study the influence of aneurysm shape and neck configuration on cerebral infarction after aneurysm operation.
Laur et al.^273^	312	Fifteen international cohorts	Absolute risk of ruptured intracranial aneurysms in growth based on triple-S prediction model.
Wojciech et al.^278^	147	Maria Sklodowska-Curie Hospital	The study identified independent predictors of surgically derived complications (residual aneurysm and cerebral ischemia) and intraoperative aneurysm rupture.
Duan et al.^279^	107	National Taiwan University Hospital	To study the clinical manifestations and genetic characteristics of patients with TAA and dissection in Taiwan.
Constance et al.^281^	415	Aneurysm-Express biobank	To investigate the association between variation in genetic susceptibility to aneurysms and clinical phenotypes including aneurysm diameter, artery type, and aneurysm-related symptoms.
Hosaka et al.^282^	862	National Clinical Database	To study postoperative survival in patients with primary aortic or common iliac artery (CIA) aneurysms treated surgically.

### Research on intracranial aneurysm

Greving et al.^255^ conducted a systematic review and pooled analysis of individual data from 8382 participants in six prospective cohort studies of subarachnoid hemorrhage to investigate predictors of aneurysm rupture. By developing a new risk scoring system for aneurysm rupture (Phase), researchers are better able to predict patients’ risk of aneurysm rupture. They observed that the average 1-year risk of aneurysm rupture was 1.4% and the average 5-year risk was 3.4%. Six factors, including age, hypertension, history of subarachnoid hemorrhage, aneurysm size, aneurysm location and geographic area, were associated with prognosis. Sex, smoking and multiple aneurysms during aneurysm detection were not helpful in predicting aneurysm rupture.^256^ The risk prediction of aneurysm rupture factors provides a good starting point for doctors and patients to discuss the pros and cons of treatment options.

Korja et al.^257^ followed 118 patients (61 women) diagnosed with unruptured intracranial aneurysms between 1956 and 1978 until death or the onset of subarachnoid hemorrhage (SAH). The median age of patients in this cohort at diagnosis of unruptured intracranial aneurysm was 43.5 years (range 22.6 to 60.7 years). The median size of unruptured intracranial aneurysms (UIAs) at diagnosis was 4 mm (range: 225 mm). The risk factors analyzed for rupture included sex, age, smoking, systolic blood pressure, diagnosed hypertension, UIA size, and number of UIAs. Subarachnoid hemorrhage occurred in 34 of 118 patients (29%). The annual rupture rate per patient was 1.6%. Female sex, smoking and an aneurysm diameter of 7 mm are risk factors for lifetime SAH.^258^ The risk value for aneurysmal subarachnoid hemorrhage ranges from 0% to 100%, and the annual rupture rate ranges from 0% to 6.5%, depending on the risk factors. Among 96 UIA patients with aneurysms less than 7 mm in diameter, 24 (25%) developed aneurysmal SAH during follow-up.^259^ This study is the first to determine the natural lifetime course of unruptured intracranial aneurysms (UIAs) and identify patients at high and low risk of rupture.

Mocco et al.^260^ analyzed the morphological features associated with aneurysm rupture by using a case‒control design in the international database of unruptured intracranial aneurysm studies. The cohort included 57 patients with ruptured aneurysms and 198 patients with unruptured intracranial aneurysms. Consistent with previous studies, larger aneurysm size^261–263^ and aneurysm location^264–266^ are important predictors of aneurysm rupture. In addition, vertical height (*P* = 0.008) and size ratio (the ratio of maximum diameter to mother vessel diameter; univariate analysis (*P* = 0.01)) were also predictors of aneurysm rupture. However, the length-diameter ratio, ascus, multilobe, aneurysm angle, cervical diameter, parent vessel diameter and calculated aneurysm volume had no statistical significance in predicting aneurysm rupture. In the multivariate analysis, vertical height was the only significant predictive factor (chi square 7.1, *P* = 0.008). Since this study is only a sample of the ISUIA cohort, its generalizability is limited. The entire ISUIA database needs to be reanalyzed to obtain more reliable results.

Goertz et al.^267^ analyzed 252 cases of ruptured and unruptured aneurysms treated with microsurgical clips from 2010 to 2018, including 148 cases of ruptured aneurysms and 104 cases of unruptured aneurysms, with an incidence of cerebral infarction of 17.1%. Further analysis found that both ruptured and unruptured aneurysms were independent risk factors for surgery-related infarction. The incidence and risk factors for cerebral infarction associated with microsurgical clipping of intracranial aneurysms have been discussed in a number of studies.^262,268–272^ Aneurysms with complex shapes are more likely to have infarction (*P* = 0.084). Similarly, the infarct rate of irregular neck aneurysms (37.5%) was significantly higher than that of normal neck aneurysms (10.1%, *P* < 0.001). In addition, irregular aneurysms of the neck were associated with higher rates of intraoperative rupture (*P* = 0.003) and temporary maternal artery occlusion (*P* = 0.037). In multifactorial analyses, irregular neck morphology was identified as an independent risk factor for infarction (*P* < 0.001), but there was no significant correlation between aneurysm shape and infarction (*P* = 0.966). The results of this study provide information on risk factors for cerebral infarction associated with microsurgical resection, which may be of assistance to neurosurgeons in their analysis and decision-making.

Laura et al.^273^ included 312 patients with growing aneurysms in the triple-S prediction model based on three independent rupture predictors (size, location, and shape) for the absolute risk of rupture of long intracranial aneurysms and found that the 1-year risk of rupture ranged from 2.1% to 10.6%. Consistent with previous studies, they also found that growing aneurysms had a higher risk of rupture than nongrowing aneurysms on subsequent imaging.^257,274–277^ The absolute risk of rupture within 1 year after growth testing was 4.3%. This study has implications for clinical practice. Once aneurysm growth is detected, preventive treatment of endovascular or neurosurgical aneurysms should be considered.

Wojciech et al.^278^ analyzed 147 patients who chose microsurgical clipping for intracranial aneurysms over a five-year period to determine the independent predictors of surgically derived complications (residual aneurysm and cerebral ischemia) and intraoperative aneurysm rupture. They found that an increase in aneurysm volume with a cutoff of 9 mm (*P* = 0.009; odds ratio [OR]: 0.644) and irregular dome shape (*P* = 0.003; OR: 4.242) were independently associated with cerebral ischemia and residual aneurysm in 13.6% and 17.3% of the cohort, respectively. Intraoperative rupture occurred in 27% of patients and was associated with patient age (*P* = 0.002; OR: 1.073), and the aneurysm volume of these patients continued to increase; the cutoff value was 7 mm (*P* = 0.003; Or: 1.205). The results of this study suggest that the risk of aneurysm rupture during surgery increases with age.

Most clinical cohort studies on intracranial aneurysms have focused on the risk assessment of different factors and aneurysm rupture. In summary, age, hypertension, unruptured aneurysm size, aneurysm location and aneurysm shape are consistently considered risk factors for aneurysm rupture. However, some analyses of some factors in different cohort studies also reached different conclusions. For example, Greving et al.^239^ excluded sex from the prediction model and believed that it had only limited predictive value. In contrast, in the clinical cohort of the Korja^241^ study, it was found that women with UIA sizes of more than 7 mm had a higher risk of life-long SAH. If women smoke and the UIA size is more than 7 mm, they will have an extremely high risk of rupture. The reason for the opposite conclusion may be that the sample size of the latter population was relatively small, and the heterogeneity of different populations was not considered in the prediction of disease progression, including gene differences, different living habits, and the use of drugs. In addition, sex is not valuable as an independent predictor in Greving’s study, while in Korja’s study, gender, smoking status and UIA size were comprehensively considered, so their interaction effect may be a reliable predictor. This suggests that when conducting clinical cohort analysis, we should try to collect patient information to exclude the influence of irrelevant variables.

The analysis of different clinical cohorts has its own limitations. In the study by Laura et al.,^257^ for example, patients with aneurysm growth detected by imaging may choose to receive prophylactic aneurysm treatment, which results in the selection of samples in the cohort favoring patients with a lower risk of rupture. It is also inevitable that researchers may miss some patients who were not diagnosed with SAH because of sudden death due to rupture or who went to another hospital after being diagnosed. These uncontrollable factors may affect the cohort analysis and should not be considered as a natural history study of aneurysm rupture risk after aneurysm growth. Furthermore, in some studies, it was not determined whether the patients had had subarachnoid hemorrhage in the past. Such patients have a relatively high risk of subarachnoid hemorrhage again, which may lead to overestimating the calculated risk.

### Other clinical cohort studies

To study the clinical manifestations of the patients with thoracic aortic aneurysm and dissection in Taiwan and genetic features, Duan et al.^279^ recruited 107 patients, including known aneurysm or dissection in 57 cases, martensite characteristics in 36 cases, members of the family of suspected aortic aneurysm or dissection in 11 cases, 3 cases of ectopic lens, and 73 cases (68.2%) diagnosed as aneurysm or dissection. Clinical manifestations and gene sequencing (NGS) were performed in all patients. Skin distention was the only phenotype significantly associated with AA or dissection (adjusted p = 0.007) of all clinical manifestations. In addition, 46 patients (43.0%) in this clinical cohort had pathogenic genes/variants, including the most common FBN1, followed by TGFBR1, TGFBR2, and FBN2. Patients with positive gene findings had higher rates of dissection than those without aortic aneurysms. This finding is very similar to the observation of Wolford et al.^280^ that patients with pathogenic variants had significantly earlier dissection than those without variants. In conclusion, this study suggests that skin dilatation may be a simple and convenient screening condition for patients with thoracic aortic aneurysm and dissection. Multigene NGS detection can not only help with early diagnosis but also suggest that diagnosed patients with aortic aneurysm may be at risk of dissection.

Constance et al.^281^ selected 415 patients with aortic and peripheral aneurysms from the thesis-Express Biobank (a biobank consisting of surgically treated aortic and peripheral aneurysms) to study the association between genetic susceptibility to aneurysm variation and clinical phenotype. The mean age was 69 ± 8.1 years, the majority (85%) were male, and 349 (84%) had been treated. The clinical phenotypes they focused on included three clinical features: aneurysm sac diameter, artery type, and aneurysm-related symptoms. Using GWAS effect estimates from previous studies, a best-fitting polygenic risk score (PRS) model was developed for each clinical phenotype. The best fitted PRS (including 272 variants, PT = 0.01015) showed a significant association with aneurysm diameter (*R*^2^ = 0.019, *P* = 0.001). There was no evidence that polygenes were associated with clinical symptoms or arterial type. In addition, 10 genome-wide significant risk variants for AAA were separately tested, and no association with any clinical phenotype was observed. The models used were adjusted for confounding factors and data normalization. If the sample size can be increased, the potential causal role of susceptibility variation in the initiation and progression of aneurysm disease can be further confirmed.

Owing to the rarity of primary infection of aneurysms in the abdominal aorta and iliac arteries, the optimal treatment strategy remains unclear because of the unknown pathologic mechanism, even though the disease is potentially life-threatening. Hosaka et al.^282^ examined the medical records of Japanese patients who underwent surgical treatment for primary infection of aortic or common iliac artery (CIA) aneurysms from 2011 to 2017. A total of 862 patients were selected from the National Clinical Database (NCD) in Japan, of which approximately 30.2% were found to be infected. The cumulative overall survival rates were 94.0, 89.7%, 82.6, 74.9, and 68.5% at 30 days, 90 days, 1 year, 3 years and 5 years after the operation, respectively. Age, preoperative shock, and hypoalbuminemia were independently associated with early- and late mortality. Once-in-a-lifetime patient replacement (EVAR) is associated with more persistent or recurrent aneurysm-related infections than open repair (P < 0.001). Propensity score matching analysis showed no significant difference between EVAR and in situ graft replacement in 3-year all-cause mortality and aortic-related mortality (*P* = 0.093 and *P* = 0.472, respectively). The study could help treat this rare disease by collecting data from a large number of patients, although it may not accurately reflect the current reality of the disease.

Clinical cohort studies of other types of aneurysms include abdominal aortic aneurysms, thoracic aortic aneurysms, and peripheral aneurysms. In these cohort analyses, in addition to the study of rupture risk factors, the analysis of auxiliary diagnosis and treatment for early clinical manifestations, the construction of a multigene risk score model and the exploration of primary aneurysm infection^266^ were also included. Limited by the number of samples and population selection, although these studies may have errors or conclusions that may not be suitable for all populations, they can still help clinical disease judgment.

In summary, an important link in the study of disease phenotypes is the construction of a clinical cohort. Medical evidence is needed for the diagnostic criteria, traceability, and prognostic research of chronic diseases.^283^ Therefore, it is important and meaningful to select more representative samples for clinical cohort studies. Research design schemes applied to different continents, races, and countries tend to draw different conclusions because the disease is influenced by external factors, their mutual influence and multiple gene regulation. If the sample is too small or comes from the same region, conclusions are often not universal. When collecting information from patients and healthy people, strict and uniform standards should be adopted, and quality management should be implemented during the study. Privacy protection and secure storage of the collected data should be considered. Clinical cohort studies can also integrate multiple studies. On the one hand, large samples of clinical data can be obtained, and on the other hand, the time and financial resources spent on data collection can be saved. At present, researchers have proposed that the combination of longitudinal cohort studies and multiomics analysis can greatly improve the effectiveness of disease research, target search and marker discovery. In addition, with the development of big data and the use of internet technology to build a network platform for clinical cohort studies, sharing data also promotes the development of clinical cohort studies.

### Therapy

At present, there is no effective drug for the prevention or treatment of AA. Although in the past few decades, studies have found that angiotensin II converting enzyme inhibitors (ACEi), angiotensin receptor blockers (ARBs) and β-blockers may have some effect on the growth of AAA.^284^ However, long-term clinical trials have shown no significant effect compared with placebo. Another statin used to target aortic inflammation is also gradually proving limited efficacy. Therefore, in clinical practice, the most effective treatment for patients with AA is still open surgical repair or endovascular aortic aneurysm repair (EVAR). Generally, when the diameter of the aneurysm is greater than 5.5 cm, surgery is required. However, due to the small diameter of the original abdominal aorta in women, treatment is often considered when it is greater than 5 cm.^285^ Open surgical treatment is performed by opening the aneurysm and placing an artificial blood vessel in it and fixing it in the normal vessel wall. In addition to midline abdominal incision to expose AAA, some chirurgeons make incisions outside the peritoneum from the left side, but whether the latter would reduce the incidence of postoperative intestinal obstruction, pulmonary complications, cardiovascular complications and fluid transfer needs further research.^286^ Moreover, because patients with aneurysm are at high risk of cardiovascular disease, the cardiac function of patients should be evaluated before surgery. Some studies have found that the perioperative mortality of AAA surgery is significantly related to the preoperative cardiac function of patients. For some patients with poor cardiac function, the mortality rate will be significantly increased.

EVAR is performed by directing blood flow through placement of a covered stent, during which the aneurysm remains intact. The stent was fixed in a segment of the normal aorta below the renal aorta and extended into the normal segment of the iliac artery.^287^ Compared with open surgical repair, EVAR is equivalent to intermediate to low grade surgery and has a lower perioperative mortality. However at the same time, the operation also has more stringent requirements for patients. First, because the stent needs to be fixed below the renal aorta, it must be ensured that there is at least 1.5 cm of normal aorta below the renal aorta as the anchoring area, and the diameter of the tumor should be within 28 mm. Second, because the stent passes through the external iliac and femoral arteries, the arteries there are large enough in diameter to accommodate the stent. For this reason, the proportion of women giving up EVAR due to the small diameter of external iliac artery is higher than that of men.^288^ Although the procedure is associated with lower mortality, the patient’s heart and other organ function should be evaluated before the procedure. For patients with AAA, the choice of surgical methods should be judged according to their own actual conditions,^37^ and clinicians should carefully consider taking more effective treatment methods on the premise of ensuring safety.

As a research hotspot in recent years, nanoparticles (NPs) have also been applied to the treatment of AA. The cell adhesion molecule α(v)β integrin, a marker of neovascularization, is a highly expressed receptor and can be used as a target of arginine-glycine-aspartic acid (RGD) peptides.^289^ Based on this, Kitagawa et al. used recombinant human ferritin with RGD (RGD-HFN) and encapsulated superparamagnetic iron oxide nanoparticles (SPION) to target AA lesions.^290^ Camardo et al. previously demonstrated that cathepsin K is overexpressed in aneurysm tissue and can be used as a potential target, so they combined matrix regenerated PEG-PLGA nanoparticles with antibodies to cathepsin K for targeted therapy of aortic aneurysms.^291^ MMP inhibitors have been widely studied as a potential treatment for AAA. However, due to low efficacy at low doses and high toxicity at high doses, an appropriate delivery system is urgently needed. The Nosoudi group delivered MMP inhibitors to AAA sites based on targeted nanoparticles to mitigate side effects in patients.^292^

Some researchers have turned to cell therapy. Wang’s team applied human mesenchymal stem cells (MSCs) in the mouse local elastase AAA model. They found that MSCs enhanced the suppressive function of Tregs and the number of Tregs in the aortic wall, thereby inhibiting the overall growth of AAA.^284^ Based on this, they also conducted a phase I Aortic aneuRysm Repression with mEsenchymal STem cells (ARREST) trial in patients, and the preliminary clinical results supported their findings.

In summary, the treatment of AA is in its infancy currently, and a large number of drugs and targeted therapies are still in clinical trials stage. There are also many drugs that have demonstrated possible therapeutic effects in mouse models that do not work in clinical cohorts. Based on current research, the treatment of AA is still surgery-based. Therefore, exploring the potential targets of AA is the main research direction of researchers in future.

## Animal model

The establishment of good animal models is helpful to elucidate the complex pathogenesis, which is used to develop new therapeutic methods or improve endovascular and surgical procedures. The same is true for aneurysm research. AA is regarded as an epidemic life-threatening disease. The first animal aneurysm models were published in the 1960s, and many other methods and models have been developed since then and have been variously upgraded and improved.^293–295^

Perhaps the most significant changes seen in human aneurysm tissue are degradation of extracellular elastic fibers and inflammatory infiltration. One of the first attempts to construct hemangiomas was to breakdown elastic fibers using protease. Anidjar et al. demonstrated the possibility of developing a rat aortic aneurysm model using porcine pancreatic elastase (PPE).^296^ Carsten et al. studied several batches of elastase and confirmed the need for inflammatory infiltration to activate macrophages, eventually causing the necessary extracellular matrix degradation in the rats, mimicking aneurysmal development in patients.^297^ The model building by Anidjar became the basis for future PPE model modifications. Periadventitial application of elastase in mice may cause similar changes and lead to the development of AAA.^298^ In addition, the calcium chloride model (CaCl_2_) is also commonly used to induce the occurrence of aneurysms and was first used in the carotid arteries of rabbits.^299^ The main symptoms induced by CaCl_2_ are the infiltration of macrophages into the middle and outer membranes and increased MMP-2/9 activity.^300^ The other most important animal model was Ang-II in apolipoprotein E (ApoE)-deficient mice, which was found to cause aneurysm development.^301^ This phenotype is associated with high rates of hyperlipidemia, vascular wall remodeling, inflammatory infiltration, and thrombosis in clinical aneurysms for male patients.^302^ In addition, animal models combining multiple drugs or physical methods have also been established.^303^

To study the etiology of aneurysms and new embolization materials, reliable aneurysm models must be established. At present, most models are focused on AAs. There are many kinds of animal models of AAA, which are relatively perfect.^304^ However, there are few animal models of TAA. In the establishment of animal models of AAA, some methods also lead to the formation of TAA, but the incidence of TAA is lower than that of AAA. Generally, AA animal models can be divided into two types, namely, nondissecting AA animal models and dissecting AA animal models. Among them, dissection AA is formed during the expansion of the aortic wall when the aortic intima opens up a gap, causing blood to enter the aortic middle layer and resulting in dissection thrombosis.

There are many common non-AA animal models, including the elastase-induced model, CaCl_2_-induced model and acellular aortic transplantation model. The animal models of dissected AA included Ang-II-induced animal models, aldosterone receptor agonist plus high salt-induced animal models and gene-edited aneurysm animal models. Models constructed by gene editing were mainly used for thoracic aortic aneurysms, including mutations of the FBN1 gene and mutations of the TGFBR1 and TGFBR2 genes.^305^

### Elastase induction model

Elastase is a proteolytic enzyme that can hydrolyze a variety of proteins, including insoluble elastin, gelatin, fibrin, hemoglobin, and albumin.^306^ In addition, elastase also has lipase and lipoprotein hydrolase activities and hydrolyzes peptide bonds with aliphatic nonpolar amino acids as carboxyl groups. Because of its strong hydrolytic activity, elastase can degrade the elastic tissue in the connective tissue of the arterial canal, resulting in damage to the arterial canal and then inducing the formation of an aneurysm model.^307^

Elastase was first used in Wistar rats in 1990 by Anidjar et al.^296^. By pressurizing PPE into the aortic segment for two hours, elastase would penetrate the inner layer, damage elastic fibers and induce arterial dilation. Nchimi et al. described in detail the two-stage process of constructing rat aneurysms using PPE.^308^ In the first stage, PPE directly damaged the elastic layer, resulting in the loss of elastic retraction force. The second stage is the appearance of white blood cells during thrombosis. Usually, within one week, aneurysms constructed by this model can reach three to four times the diameter of the original aorta.^309^ However, in some cases, aneurysm rupture is less common because of the large amount of elastic fiber degradation. Moreover, simple elastin may not induce successful aneurysms due to the presence of pollutants in the preparation.^310^

In addition to rats, Pyo et al.^311^ used mice to study the different roles of MMP-9 and MMP-12 in the destruction of medial elastic fibers after initial aortic injury caused by PPE use. Molacek et al.^294^ compared different aortic aneurysm induction techniques using pigs as experimental models and found that intravascular infusion of PPE caused aortic dilation with inflammation and destruction of the elastic lamina.

### Calcium chloride (CaCl_2_)-induced model

Gertz et al.^299^ surgically cut the carotid sheath of male rabbits and treated the surface of the outer membrane of the common carotid artery with 0.5 mol/L CaCl_2_ calcium chloride solution and found progressive focal aneurysm dilation, endometrial fibromyosis, calcium deposition, and rupture of middle elastic tissue. The media tissue was disturbed and infiltrated by inflammatory cells. Chiou et al.^312^ successfully induced aneurysms in mice by using CaCl_2_ in 2001. Aortic aneurysm formation was observed in C57BL/6 mice treated with 0.68 M CaCl_2_ for 10 min and three weeks later. Histological examination also showed aortic dilation with VSMC depletion, elastin degradation, and lymphocyte and macrophage infiltration. High concentrations of proinflammatory cytokines and MMPs were also detected in the dilated aorta.^313,314^ At present, the specific mechanism by which CaCl_2_ induces aneurysms is not clear, but it is known that calcium ions have a high affinity for elastin. When treated with CaCl2, cells will transport soluble ionized calcium into the cell, and VSMC alkaline phosphatase will further convert it into calcium phosphate (CaPO_4_) and precipitate it on the elastin network. Elastic fibers are damaged.^68^

### Acellular aortic transplantation model

Allaire et al.^315^ found that arterial homografts using sodium dodecyl sulfate (SDS) treatment can prevent expansion and reduce inflammatory infiltration, keep the inside of the outer membrane of elastin, and process allogeneic grafts with a uniform distribution of inflammatory intimal thickening. Their elastic protein fiber content is higher than that of untreated allograft grafts. The researchers took guinea pigs’ inferior renal aortas, treated them with SDS solution and decellularized them. Decellularization resulted in the loss of smooth muscle cells from the graft, while elastin and collagen networks remained intact. The aorta was then orthotopically transplanted into Lewis rats. The acellular aorta thus became the target of an immune response, leading to degradation of the extracellular matrix and progressive arterial dilation, forming a piriform aneurysm. Aneurysms and collagen-rich intracavitary thrombi began to form within 3–4 weeks of implantation.

This aneurysm model can be used to observe the role of ECM immune-driven proteolysis and adaptive immunity. In addition, this model can also be used to study the protective effect of homologous VSMCs and endothelial cells on the development of abdominal aortic aneurysm, as well as the ability of VSMCs to inhibit MMPs.^315^ The model can also be used to study the effects of serine and MMPs and their inhibitors.

### Ang-II-induced model

Earlier studies have found that AAA occasionally occurred in C57BL/6 mice after subcutaneous infusion of Ang-II. Daugherty et al. first reported in 1999 that subcutaneous infusion of Ang-II with a slow-release pump at a dose of 0.5 or 1 μg/min/kg for 28 days induced AAAs in ApoE-/- mice.^316^ Since then, hundreds of studies have used this model, which is the most commonly used modeling method for chemically induced AAA at present. Ang-II infusion mimics AAA inflammation and promotes suprarenal aortic dilatation, atherosclerosis in hypercholesterolemic mice, macrophage accumulation, and thrombosis.

Compared with transgenic mice with congenital dyslipidemia, C57BL/6 mice have a much lower incidence of Ang-II-induced AA. Studies have shown that AAA can be induced in only ~10–20% of C57BL/6 mice after Ang-II infusion. This limits the application of the Ang-II model. To augment Ang-II-induced AAA in C57BL/6 mice, one approach is to increase plasma cholesterol by inducing a PCSK9 gain-of-function mutation in mice on a C57BL/6 background.^317^ Another popular modification^318^ is to use Ang-II in combination with β-amino-propanitrile (BAPN). Male C57BL/6 mice were subcutaneously infused with Ang-II with a sustained release pump for 4 weeks at a dose of 1 μg/kg/min, and BAPN was added to drinking water at a dose of ~1 g/kg/d. This approach leads to a high incidence of aortic rupture.

### Aldosterone receptor agonist induced by high salt intake

Deoxycorticosterone acetate is a precursor of aldosterone and a ligand of glucose and melanocortin receptors. Liu et al.^319^ induced AA models by administering deoxycorticosterone acetate (DOCA) and high salt to 10-month-old male C57BL/6 mice over a three-week period. AAs induced using this model were similar to human AAs in terms of elastin degradation, inflammatory cell infiltration, VSMC degeneration and apoptosis, and oxidative stress. Wu et al. found that p-selectin glycoprotein ligand-1 (PSGL-1) promoted the formation of DOCA salt-induced aneurysms in mice, and PSGL-1 knockdown significantly reduced the aneurysm formation rate and mortality in mice. Further studies found that PSGL-1 knockdown inhibited the adhesion of white blood cells and ECs and then inhibited the infiltration of inflammatory cells and the expression of inflammatory factors,^320^ indicating the important role of the inflammatory response in salt-induced aneurysms.

### Gene editing

Aneurysm models constructed based on gene editing are mainly animal models of TAA because genetic factors are important factors for the occurrence and development of TAA. Under the condition of specific gene mutation, the pathogenesis of TAA is mainly attributed to the degradation of ECM, contractile dysfunction of VSMCs and dysfunction of the TGFβ signaling pathway.^321^ More than 20 gene mutations have been associated with the occurrence of TAA and dissection.

FBN1 encodes fibrin, an ECM glycoprotein, whose mutation was first identified in Marfan syndrome. Approximately 75% of patients with the disease have TAA and dissections, so FBN1 was first identified as the gene responsible for TAAs. Pereira et al. constructed mice with TAA and dissection by knockout of the FBN1 gene, but this method was not suitable for model construction because the born mice died of cardiovascular complications 10~14 days later.^322^ However, by mutating cysteine 1039 on FNB1 protein into glycine (C1039G), heterozygous mutant mice with normal life spans were created, and lesions in the middle vascular layer of mice gradually appeared two months after birth. This model was also gradually applied to the study.

Mutations in TGFBR1 and TGFBR2, the receptors for TGF-β, cause Leudiz syndrome. Leuditz syndrome is similar to Marfan syndrome in terms of the phenotype of cardiovascular disease, but in the former, it has a faster rate of aneurysm expansion. Direct knockout of the TGFBR1 and TGFBR2 genes in mice or specific knockout of the TGFBR1 and TGFBR2 genes in VSMCs can lead to embryo death.^323^ Li et al.^324^ used tamoxifen to induce specific knockout of TGFBR2 in VSMCs after mouse birth, which led to TAA. In addition, the mice with Tgfbr1M318R/+ and Tgfbr2G357W/+ heterozygous mutations also developed TAA and dissection, and death began to occur two months after birth.

Although there are a variety of ways to build an aneurysm model, each has certain limitations and advantages. For example, if there are pollutants in the elastin used in the elastase-induced model, the aneurysm will not be induced. The aldosterone receptor agonist plus high salt-induced model requires 10-month-old elderly mice. In Ang-II-induced models, aneurysms mainly occur in the superior renal aorta or thoracic aorta, while the most commonly affected site in human patients is the inferior renal aorta. Animal models of aneurysms constructed by gene editing technology often require a long time of hybridization and matching, which is relatively difficult to obtain. Therefore, the selection of the model should be based on the actual experimental requirements and conditions. After construction, the models were evaluated by morphometry, histology, immunohistochemistry, Western blotting, and quantitative real-time PCR. For example, a relative dilatation rate of the abdominal aorta above 1.5-fold change was defined as AAA. Morphological assessment was performed using a grading scale. Elastin degradation in the aorta was scored in a double-blinded manner by staining sections of the abdominal aorta with hematoxylin eosin and Elastin Van Gieson (EVG). Western blotting and ELISA can be used to detect related proteins, and real-time PCR can be used to analyze mRNA differential expression. High-resolution ultrasound provides a detailed morphological assessment that can accurately define the size of AAA, the presence of intramural hematoma, and luminal thrombus, as well as assess biomechanical properties of the vessel wall, such as perimeter strain or pulse wave velocity.^325^

**Fig. 3:**
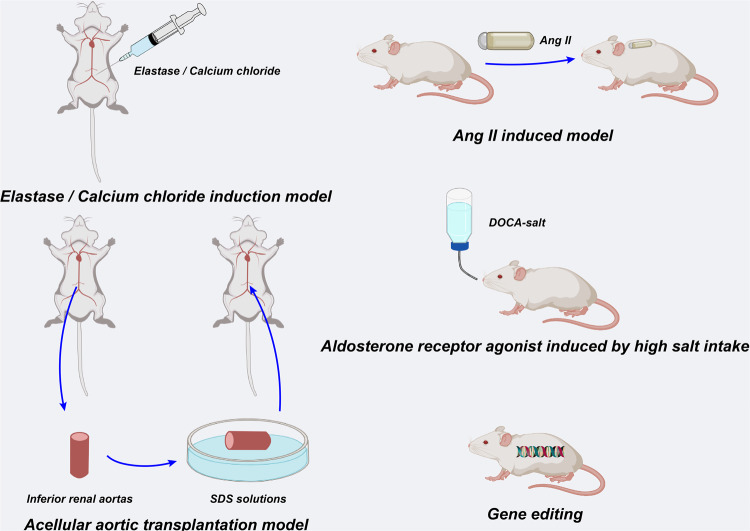
The main animal model of AA

It is a conventional and most commonly used method to construct aneurysm models using rodents such as mice and rats. However, due to the large differences between the structures of rodents and the human body, it is difficult to directly translate the corresponding mechanism studies into clinical practice. More animal models that can relatively accurately simulate the occurrence and development of human aneurysms are needed, which will require larger animals or improvements to the existing rodent model construction methods to better provide a basis for disease research (Fig. 3).

## Conclusions

Throughout the history of aneurysm research, from the initial definition of aneurysm to the exploration of the pathological characteristics and etiology of aneurysm to the in-depth molecular mechanisms of the aneurysm formation process, researchers have progressively developed their understanding of aortic aneurysms. However, how to set up a standard protocol to screen aneurysms earlier, how to treat the progression of the pathologies, and how to prevent aneurysms from rupturing remain to be resolved.

To prevent AA, we believe that researchers should enhance the combination of clinical medicine and basic medicine and conduct multiomics assays in patient samples of different clinical typologies. By combining deep learning and artificial intelligence, researchers could find the expression profiles of genes, proteins, metabolism and other characteristic substances in AA patients of different ethnicities. Therefore, we could perform routine aortic diameter monitoring in populations with susceptibility gene mutations or high levels of expression of pathogenic proteins or metabolites, and better assays of these targets could be developed to monitor AA in the form of kits or rapid tests. Inspired by the features of abnormally expressed pathogenic proteins in AA tissues, targeted molecular probe techniques should be developed to help quantify the aortic diameter and rupture risk of AA via noninvasive imaging. Moreover, AA-related science education is needed for high-incidence populations such as older men with smoking habits.

To treat AA, researchers should find drug treatment targets, such as proteins and metabolites that participate in VSMC phenotypic switching or inflammation progression, by multiomics assays in AA patients. Moreover, the development of smart drug delivery systems provides a good alternative to highly efficiently inhibit AA progression. For researchers, finding animal models that are faster, cheaper, and more closely resemble the state of AA in human patients is needed. Overall, more studies are needed to further explore the definitive mechanisms related to AA in the coming decades.
